# Sexual functioning in 4,418 postmenopausal women participating in UKCTOCS: a qualitative free-text analysis

**DOI:** 10.1097/GME.0000000000001377

**Published:** 2019-07-08

**Authors:** Helena Harder, Rachel M.L. Starkings, Lesley J. Fallowfield, Usha Menon, Ian J. Jacobs, Valerie A. Jenkins

**Affiliations:** 1 Sussex Health Outcomes Research and Education in Cancer (SHORE-C), Brighton and Sussex Medical School, University of Sussex, Brighton, United Kingdom; 2 MRC Clinical Trials Unit at UCL, Institute of Clinical Trials & Methodology, University College London, London, United Kingdom; 3 EGA Institute for Women's Health, University College London, London, United Kingdom; 4 University of New South Wales, Sydney, Australia.

**Keywords:** Ageing, Free-text analysis, Postmenopausal, Qualitative research, Sexual activity, UKCTOCS

## Abstract

**Objective:**

Sexual well-being can contribute significantly to the overall quality of women's lives. This qualitative study aimed to examine sexual activity, functioning, and satisfaction in a large sample of postmenopausal women from the UK Collaborative Trial of Ovarian Cancer Screening (UKCTOCS)

**Methods:**

Thematic analysis was used to evaluate the free-text data of the Fallowfield Sexual Activity Questionnaire (FSAQ) completed by UKCTOCS participants at baseline before annual screening.

**Results:**

A total of 24,305 women completed the baseline FSAQ and 4,525 (19%) provided free-text data, with 4,418 comments eligible for analysis. Median age was 64 years; 65% had a partner and 22.5% were sexually active. Four interrelated themes were derived: partner availability, physical and sexual health, mental well-being, and interpersonal relationships. Primary reason for absence of sexual activity was lack of a partner, mainly due to widowhood (*n* = 1,000). Women discussed how partner's medical condition (27%) or sexual dysfunction (13.5%), their own physical health (18%) or menopause-related symptoms (12.5%), and prescribed medication (7%) affected sexual activity. Impact of low libido in self (16%) or partner (7%), relationship problems (10.5%) or logistics (6%), and perceptions of ageing (9%) were also mentioned. Few (3%) referred to positive sexual experiences or had sought medical help for sexual problems (6%).

**Conclusions:**

This qualitative analysis explored postmenopausal women's perspective on their sexual functioning. Having an intimate partner and good physical health are key factors for continuation of sexual activity and satisfaction. Further sexual education for healthcare professionals is needed to raise awareness about sexuality and sexual difficulties in later life.

## Abstract 2

Supplemental Digital Content is available in the text

## Abstract 3

***Video Summary*:** Supplemental Digital Content 1.

Much research has been conducted into the biological reasons why sexual function, intimacy, and sexual satisfaction decrease in women after menopause, in particular the hormonal and physical changes (ie, vasomotor symptoms, VMS), urogenital changes (ie, vaginal dryness and painful intercourse), sleep disorders, or metabolic disorders.^1-8^ Other factors such as psychosocial changes (body image concerns, self-confidence and desirability, stress, mood changes), sociocultural influences (cultural and religious beliefs about sexuality), and relationship characteristics (communication, satisfaction, partner's sexual function), however, also play an important role.^4,9-12^

There is less research about sexuality and ageing from the perspective of older women, with limited qualitative analysis of sexual activity and satisfaction. A recent systematic review identified 20 qualitative research papers about older people's attitudes and concerns about sex and sexuality in later life.^13^ The majority were published in the last 10 years (70%) and involved both men and women (65%). The studies used semistructured or in-depth interviews only; free-text data from surveys or validated questionnaires were not included. Many health or sexual questionnaires include a space for the addition of these observations to complement the usually closed-ended questions. These comments provide additional context and narrative around individuals’ experiences. This added value is, however, often overlooked and the qualitative extracts are regularly excluded from analysis.^14,15^

The purpose of this paper is to present a qualitative analysis of the free-text comments from a unique data set on sexual activity collected in the UK Collaborative Trial of Ovarian Cancer Screening (UKCTOCS). The primary aim of this large multicenter trial was to assess the impact of different forms of ovarian cancer screening on disease mortality.^16^ Secondary objectives include measuring the effects of screening on anxiety levels, psychological morbidity, and sexual activity, and have been published previously.^17-19^

The UKCTOCS sexual activity data showed that at baseline, before the start of annual screening, approximately half of women were sexually active. A decrease in all aspects of sexual activity was observed across time: sexual activity was less frequent, not as pleasurable and more uncomfortable. These findings were, however, not associated with ovarian screening, except for women with abnormal results who underwent repeated or higher level screening.^19^

The current evaluation explores self-reported sexual activity and functioning in a subset of UKCTOCS participants. Such qualitative analysis of free-text data provides a unique opportunity to explore the perspectives of a very large number of UK women about sexual functioning, intimacy, and sexual dysfunction. These findings may be of value to others conducting research about the impact of disease and treatment on patients who often lack a control group.

## METHODS

### Study design and participants

Full details of the UKCTOCS study design have been described elsewhere.^17,20^ In brief, a total of 202,638 postmenopausal women (aged 50-74) were recruited in 13 UK trial centers and randomized to either annual ovarian cancer screening with serum CA 125 or transvaginal ultrasound, or no intervention (control group). Of these, 185,693 (91.6%) women participated in a psychosocial study (nested within UKCTOCS) and completed a series of questionnaires at study entry before randomization, including the Fallowfield's Sexual Activity Questionnaire (FSAQ).^21^ FSAQ is a widely used validated questionnaire to measure self-reported female sexual activity and functioning.^22^ There are three sections assessing if a woman is sexually active (section I), probing reasons for lack of sexual activity (section II), and measuring sexual pleasure (eg, desire, enjoyment), discomfort (vaginal dryness, dyspareunia), and sexual habit (section III). There are also free-text questions where participants can provide additional comments.

A complete psychosocial assessment of >185,000 women in UKCTOCS for 6 years was not feasible; therefore, a partial longitudinal follow-up on a cohort of 26,167 women was conducted. The cohort included a group of randomly selected women from each of the three study groups, and any women in the two screening groups who were recalled for a repeat clinical screen after an abnormal test result (a psychosocial study consort diagram was published previously)^17^. The questionnaire data of these 26,167 women were entered in the study database, including 24,305 (92.9%) who completed baseline FSAQs (Table 1). The free-text data of the baseline FSAQ was used for this qualitative analysis. Ethical approval for the trial was obtained from the UK North West Multicentre Research Ethics (MREC 00/8/34). All participants provided written informed consent at study entry.

**TABLE 1:**
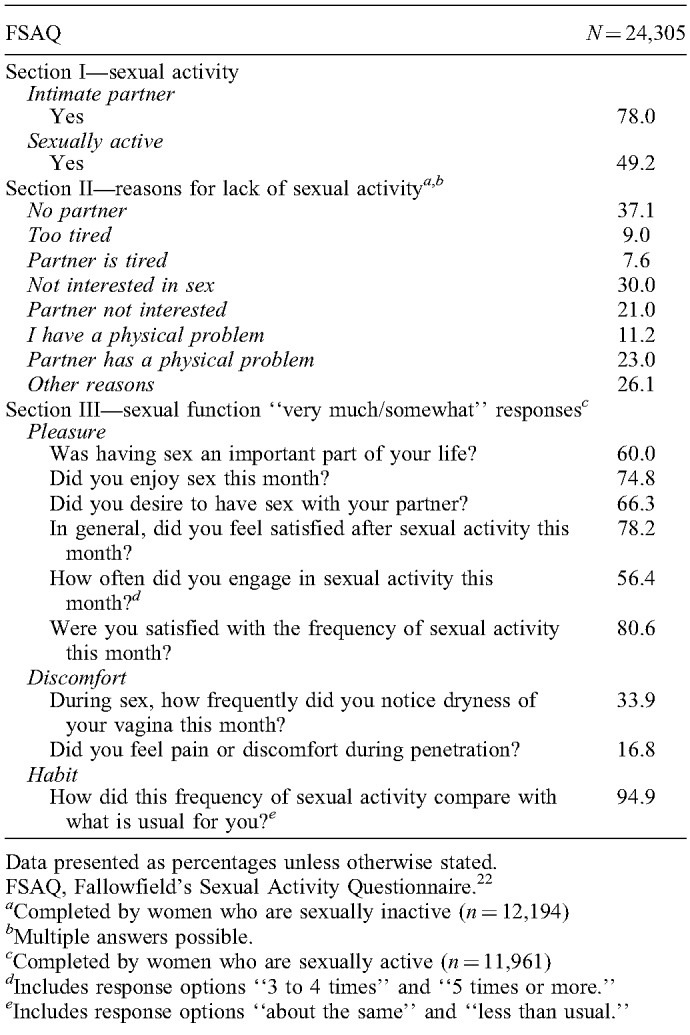
The results of FSAQ at study entry

### Data analysis

Participants’ free-text comments in the FSAQ along with the demographic survey data were used for the purposes of this analysis. All identifiable information was removed at data-entry. The comments were analyzed independent of the FSAQ quantitative results using thematic analysis with a coding framework developed by three researchers (VAJ, RMLS, HH).^23,24^ The content of the comments was coded and grouped into themes and subthemes. The initial coding scheme included some predefined response options of the FSAQ. There was also a code for comments that were entirely factual (eg, “no sexual activity for many years,” “married”), irrelevant or miscellaneous (eg, “don’t know”), or related to the content of the questionnaire (eg, “questions not acceptable,” “too personal”); these responses were excluded from further analysis. After initial coding highlighted relevant discussion themes, all text segments were iteratively analyzed. To structure the large data set, responses were grouped into whether it was related to unavailability of an intimate partner, physical/mental health, general well-being, or psychosocial factors. Responses were given as many codes as appropriate to cover the content of the comment. The coding frame was refined and themes were added or merged until they effectively represented all text segments. Any discrepancies or disagreements were discussed by the team and adjustments made if necessary. Microsoft Excel was used to sort and structure the qualitative data. The key findings are organized under headings that relate to the main themes from the free-text data.

Descriptive statistics were used to summarize quantitative data. Associations between variables were evaluated using Student's *t* tests and χ^2^ tests. A *P* value ≤0.05 was considered significant. The quantitative analysis was facilitated by IBM SPSS Statistics 25.0 (IBM Corp, Armonk, NY).

## RESULTS

The baseline FSAQ results for 24,305 women who were included in UKCTOCS's longitudinal follow-up are displayed in Table 1. A total of 4,525 (18.6%) women made free-text comments in sections II or III of the FSAQ. Of these, 107 (2.4%) responses were excluded because they were irrelevant, miscellaneous, or referring to the questionnaire content. The comments of the remaining 4,418 women were analyzed. Participants’ characteristics are shown in Table 2. Median age was 64 years. A total of 2,883 (65.3%) women had an intimate partner, and 995 (22.5%) reported that they were sexually active (indicated by the term “active”). Partnered women and sexually active women were on average 2.9 and 4 years younger (*P*<0.001).

**TABLE 2:**
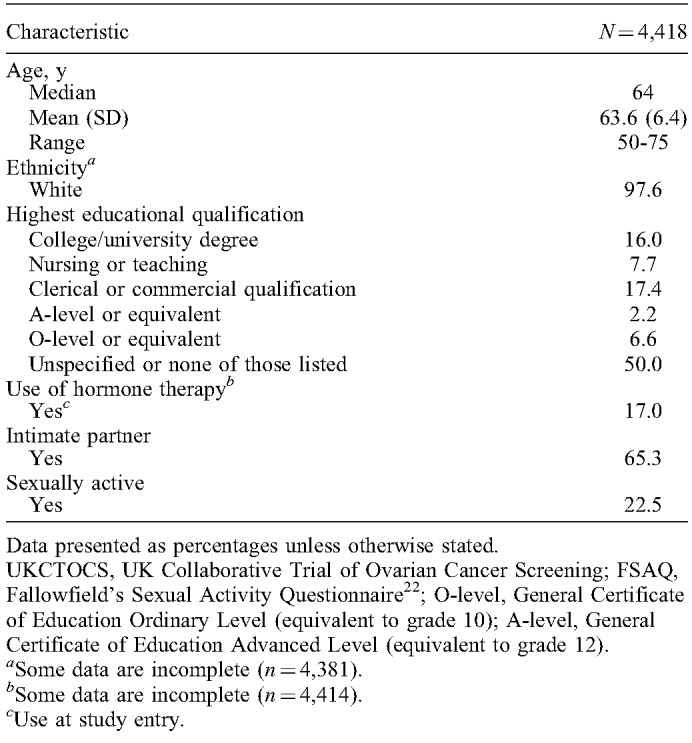
Characteristics of UKCTOCS participants who used the free-text box of the FSAQ at study entry

The demographic characteristics of women who made free-text comments were compared with those who did not. Women who provided comments were on average 2.5 years older (*P* <0.001), were less likely to have an intimate partner (65.3% vs 81.5%; *P* <0.001), and had lower levels of sexual activity (22.5% vs 54.9%; *P* <0.001). Small differences in education were also observed (ie, lower proportions of clerical/commercial/O-level qualifications, and a higher proportion of unspecified/unlisted qualifications in women who provided free-text comments; *P* <0.001).

Free-text comments ranged from one word or one sentence to longer phrases. The majority of comments (3,426/4,418; 77.5%) were made in section II of the FSAQ which probes the reasons for sexual inactivity. Most comments (3,496/4,418; 79.1%) were assigned a single code; 12.5%, 6.2%, and 2.2%, respectively, were allocated 2, 3, and 4 codes. Representative quotes illustrating the main findings are included in Table 3.

**TABLE 3:**
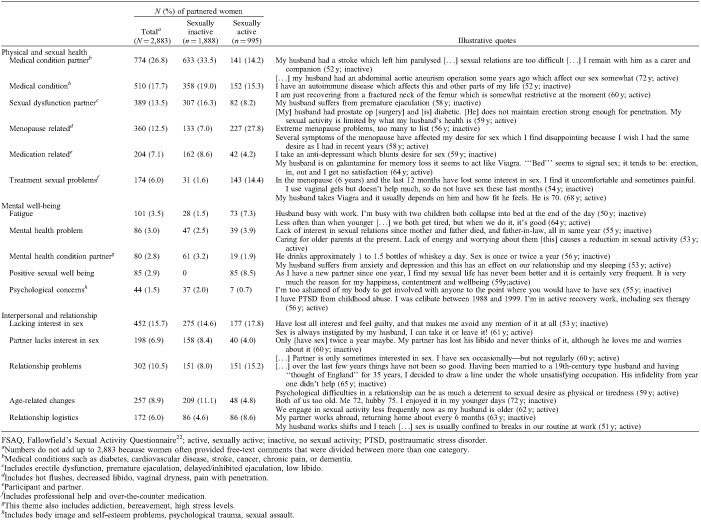
Main themes and subcategories derived from the free-text data on the FSAQ of partnered women

### Partner availability themes

The majority of women (3,423/4,418; 77.5%) were sexually inactive (indicated by the term “inactive”), mainly due to lack of an intimate partner (1,535/3,423; 44.8%). Most were widowed (998/3,423; 29.2%) and mentioned that they found it difficult to meet other men, had lost interest in sex after their partner's death, or did not want to engage in other sexual relationships: “I have been a widow for 17 years. My husband was my childhood sweetheart, there will never be anyone else” (72 y; inactive).

Some women said that they were inactive because of a separation or divorce (145/3,423; 4.2%). A small number (81/3,423; 2.4%) cited they were celibate, usually because of religious reasons. Some explained that lack of partner and sexual activity were temporary due to caring responsibilities for older parents or children: “There is no sexual activity in my life at present because I do not have a partner and I feel my role in life at present is to bring up my 12-year old son, relationships come second” (50 y; inactive).

Several women indicated that they were satisfied living without a partner and they could be described as happy singles: “I have been on my own for 18 years, therefore without sex. I don’t miss sex, I don’t think about it, and I am quite content leading a single life” (51 y; inactive). Others, however, found it much harder to cope with living a single life, or mentioned that they had lost the confidence to start (new) relationships: “I have found it very difficult to meet a man since I have been divorced. This does make me sad as I would love a good friend” (64 y; inactive).

### Physical and sexual health-related themes

Table 3 shows the physical health-related themes derived from comments of women with an intimate partner (*n* = 2,883), including medical condition (self, partner), partner's sexual dysfunction, menopause, medication, and treatment of sexual problems.

Medical conditions or poor health of partners was repeatedly mentioned (774/2,883; 26.8%), mostly by sexually inactive women (633/774; 81.8%). Sometimes multiple medical conditions and complex care needs were reported: *“*My husband has Parkinson's disease and TB of the vertebrae. At the age of 77 he also has dementia and is in hospital at this present time due to a fall” (73 y; inactive).

Women also reported that their own health-related problems (510/2,883; 17.7%) impacted sexual activity and satisfaction. Some problems were associated with menopause (360/2,883; 12.5%), including vaginal dryness, painful intercourse, reduced libido and arousal, and difficulty achieving orgasm. Women usually mentioned multiple issues as disruptive to their sex life: *“*Since the menopause, an extremely important part of my life, intercourse, is ruined. This is because of vaginal dryness and spasm, reduction in physical desire (but not mental), and change and huge reduction in gaining orgasm and in intensity of orgasm” (55 y; inactive).

Sexual dysfunction of the intimate partner was mentioned by 13.5% (389/2,883) of participants. This predominantly involved erectile dysfunction (ED) which had limiting effects on women's sexual satisfaction. ED often related to chronic medical conditions such as heart disease, obesity or diabetes. In some cases psychological factors interfered with sexual feelings and resulted in ED: “My husband has a very stressful job and when we make love he has a problem with keeping his erection long enough to satisfy us both” (57 y; inactive).

Sexual problems were sometimes linked to medication use, both for women and their partners (204/2,883; 7.1%). Most responses referred to drug-related loss of libido, ED or the inability to have penetrative sex. Few women mentioned that these problems had been restricting their sex life for many years, whereas others had accepted the situation: “My husband is on medication which prevents him from getting an erection. However we are both quite happy with a kiss and cuddle after 42 years of marriage” (64 y; inactive).

Many of these physical health-related problems had significant effects on sexual function. Some women reported that they or their partner were embarrassed and reluctant to discuss sexual issues with a healthcare provider: *“*My husband is taking tablets which may or may not make him impotent. At 75 he thinks it's not necessary to discuss that with his GP [primary care physician], I disagree” (68 y; inactive).

Help seeking and treatment for sexual problems were only mentioned by 174 participants (174/2,883; 6%), predominantly by sexually active women (143/174; 82.2%). A primary healthcare professional, usually a primary care physician, was the main source of support for participants who disclosed this information. Erection-enhancing medication (ie, sildenafil) was often mentioned in relation to ED. Interventions used for menopause-related symptoms were mostly vaginal lubricants or moisturizers and hormone creams. Current or past use of systemic hormone therapy (HT) was mentioned by 63 women (2.2%). Few women described how treatment for menopausal symptoms had a positive influence on their general well-being: *“*The menopause and tiredness has been affecting me badly. Began HT this month and feel more positive that doing something about it with great support from my GP [primary care physician], friends and family” (50 y; active).

Other women had developed their own coping strategies, altered their views toward sex within a relationship, and emphasized the importance of love, companionship, and commitment. Some adapted their sexual behavior or found alternatives for sexual intercourse (eg, masturbation, oral sex) which enabled them to continue to have an intimate relationship: *“*My husband has heart problems so we don’t have sex a lot. We satisfy each other in other ways which is fine with me” (62 y; active).

### Mental well-being themes

Several mental well-being-related themes were noted: mental health conditions (self, partner), fatigue (mental, physical), psychological concerns, and positive sexual well-being (Table 3). Mental health issues, including depression, anxiety, bereavement, alcohol addiction, or stress were mentioned by some women (166/2,883; 5.8%), and affected women and partners equally. The mental health condition or associated treatments often caused reduced libido or ED: *“*My husband takes Venlafaxine anti-depressant which renders him incapable of maintaining an erection. At present he is being weaned off these tablets and will start a different sort. Hopefully then our sex life will get back to normal” (55 y; inactive).

Work-related stress or stress associated with the demands of caring for older relatives was also mentioned in this sample: “Caring for older parents at the present. Lack of energy and worrying about them causes a reduction in sexual activity” (53 y; active).

Physical (and mental) fatigue was another reason for low sexual activity (101/2,883; 3.5%). This was mainly reported in sexually active women (73/101; 72.3%) and often related to work, caring duties, or juggling several responsibilities at the same time. Some indicated that the fatigue was temporary and related to pleasant disruptions in their daily life: “The arrival of a guide dog puppy […] which wakes at 5.30 am left two adults feeling tired each evening!” (61y;active).

Psychological concerns were mentioned by very few women (44/2,883; 1.5%), and included traumatic experiences, phobias, low self-esteem, or problems related to physical appearance, body image, or self-perception: “I had breast cancer and feel less feminine with the scars and deformity” (57 y; inactive).

A few comments referred exclusively to positive sexual experiences or age-related changes (85/2,883; 3.0%). Some of these women noted reduced frequency or loss of spontaneity, but highlighted that other aspects, such as nonpenetrative sex, had become more important. Others highlighted their satisfaction with their sex life and relationship: “We are in our 60s so quality rather than quantity matters. I have a problem with vaginal dryness so we have to be very imaginative! I am married to a lovely man” (59 y; active).

Few reported that their sexual activity was as good as it had ever been in their relationship, or mentioned that a new partner positively influenced their sexual well-being: “As I have a new partner since one year, I find my sexual life has never been better and it is certainly very frequent. It is very much the reason for my happiness, contentment and wellbeing” (59 y; active).

### Interpersonal and relationship themes

Table 3 shows the interpersonal themes, including lack of interest (self, partner), and age-related or logistical relationship problems. Approximately 16% of women (452/2,883) and 7% of partners (198/2,883) lacked interest in sex. Sometimes couples made a mutual decision to stop activities but not always: “I would still welcome an active sex-life, but my partner who is 57 years old does not seem to have the same sex drive as he did before his fifties. So sex only happens once every 3-4 months” (55 y; active).

Occasionally, women indicated that sex was central to a committed relationship. Some perceived sex as an act toward benefiting their spouse rather than themselves engaging in activity despite pain or lack of pleasure: “I indulge in sexual activity to please my husband. I do not feel any sexual satisfaction from the activity. I do however enjoy the closeness and I am pleased that my husband enjoys the activity” (67 y; active).

Additional comments about relationship difficulties were made by just over 10% of women (302/2,883). Understandably, these problems frequently had a negative influence on sexual activity, sometimes for many years: “Married over 36 years, going through another ‘sticky patch’. No third party involved by either side, but arguments put me off sex” (58 y; inactive).

Around 9% of (predominantly sexually inactive) women (257/2,883) referred to age in the free-text comments. Most responses reflected how they accepted lack of sex as part of a natural course of ageing. Sexual desire and the importance of the physical aspects of sex (intercourse, orgasm) decreased, whereas closeness and connection had become more important. Some women mentioned how commitment and care were key elements in their relationship, especially if the partner had a medical condition: “He is 82 and has lapses of memory. We have had a very good relationship in the past. What is required now is tolerance, understanding and compassion.’ (72 y; inactive).

The logistics within relationships also played a role in reduced sexual activity. Some women (172/2,883; 6%) said sex was infrequent due to shift-work, lack of privacy, or because their partner worked abroad or lived elsewhere: “Low quantity due to geographical distance from partner not lack of desire! Have high libido and have to masturbate every day! (I can’t believe I’m telling you this!)” (52 y; active).

## DISCUSSION

Sexuality remains an important aspect of ageing with international research showing that despite common misconceptions, a large proportion of older adults stay sexually active.^1,25,26^ Women at all ages, however, report less activity than men, and in later life around half of them have sexual dysfunction.^25,26^ The present study analyzed 4,418 free-text comments from a sexual activity questionnaire (ie, FSAQ) in women aged 50 to 75 years who participated in a large clinical trial, to examine self-reported sexual functioning. Our data showed notably lower levels of sexual activity with just under a quarter (22.5%) of all women reporting that they had intercourse in the preceding month.

The most cited reason for being sexually inactive was lack of an intimate partner (34.7%) predominantly due to widowhood. A study in 2,374 community-dwelling adults aged 65 or older found that the lack of a partner was the greatest barrier to being sexually active at older age.^27^ Only 5% of unpartnered women in this study engaged in physical tenderness (fondling, kissing) and just over 1% reported having sexual intercourse. It was clear from our data that many older women restricted sexual activity to committed relationships. Sometimes they were unable to find new partners after a relationship break-up or after the death of a spouse. The loss of a partner is often experienced as one of the most stressful life events in later life, and it is not surprising that many women abstained from sex or were reluctant to engage in new intimate relationships.^28^ Concerns about remaining loyal to the deceased partner, losing someone again, or having trepidation about opening up in a relationship influence the formation of new relationships in widowhood and enhance the tendency to avoid intimacy and sexual activity.

Levels of sexual activity in partnered women in our study were lower than published data. Around a third of women with an intimate partner (34.5%) said that they had been sexually active in the preceding month. Sexual functioning was strongly related to poor physical health of the intimate partner or women themselves, partner's sexual problems, and menopause-related symptoms, particularly vaginal dryness and dyspareunia. Interpersonal and relationship factors also played a role. Mental health and psychological concerns affected sexual activity to a lesser extent, although it is possible that some women were more reluctant to report these problems. Overall the current findings support previous research on the role of physical and mental health in understanding sexual problems experienced by older women.^25,26,29,30^

Medication is also a common contributor to sexual dysfunction; 7% of partnered women mentioned that drugs for physical or mental health conditions affected their sex life, and the majority had stopped sexual activity as a result. It is recognized that significant numbers of older people take various drugs, some of which are known to influence sexual functioning.^31^ A UK health survey showed that nearly half of all adults had taken at least one prescribed medicine in the last week, and almost a quarter had taken three or more.^32^ Weekly prescribed medicine use increased with age to 90% of those aged 75 or over, and polypharmacy is often an important factor in lack of sexual activity. Women in our study regularly referred to medication for blood pressure, cholesterol levels, pain, and depression. These drugs are frequently prescribed in the ageing population, and evidence has shown that they and their antecedent diseases may impact negatively on sexual function.^31,33,34^

The findings presented here also confirm that reduced sex drive in women or partners influences sexual functioning and satisfaction. Loss of libido is not uncommon in older women. Previous studies have highlighted that prevalence of reduced libido tends to increase with age and is associated with poor physical or mental health, menopause-related hormonal changes, and having a partner with sexual problems; all of which were reported in the present study.^30,35-37^ It can, however, also be the result of ongoing interpersonal issues, such as a lack of connection with the partner, unresolved conflicts, or poor communication about sexual needs and preferences. Our data showed that 1 in 10 women experienced relationship problems or conflicts with partners, which may have influenced their activity levels.

Not all women in the present study reported negative changes or expressed concerns about their sex life; a small minority (3%) reported optimistic and positive sexual experiences. These women described that they enjoyed sex in later life, and they were satisfied with their sexual relationships and frequency of activity. Earlier research has shown that positive attitudes toward sex, sexual changes related to ageing, and relationship happiness were significant predictors for sexual activity and sexual intercourse.^26,35^ A recent qualitative study in older men and women highlighted that sexual activity helps to maintain overall functioning, and make older adults feel “young again,” attractive, and desirable.^38^

In contrast, sexually inactive women often emphasized the importance of the emotional aspects of their relationship, such as tenderness and affection. They referred to good (sexual) memories and described how they had lowered their expectations, or had redefined their marriage by becoming companions instead of lovers. These findings show that women adapt to negative sexual changes by altering their attitudes and behavior, and prioritizing different aspects of sex, such as nonpenetrative sex or fondling, kissing, and cuddling. This is consistent with previous research on sexual changes during the transition to later life.^36,39-41^

Few older women were seeking medical help for sexual difficulties. Just 6% said that they or their partner had discussed sexual issues with a healthcare professional (HCP) or used drugs to relieve symptoms, including over-the-counter products such as lubricants. Poor help-seeking behavior for sexual dysfunction among older people has been demonstrated in previous studies, including qualitative research.^42-47^ In one study in women diagnosed with vulvar or vaginal atrophy, 72% never had a discussion with a HCP about their symptoms.^46^ Perceived barriers to seeking help include feelings of embarrassment, discomfort, or failure, but also beliefs that “sex is private” or that sexual problems are part of normal ageing or “something to live with.”^37,39,46,48^ In addition, HCPs’ attitudes toward sexuality in older people often influence the perception and management of sexual problems. HCPs frequently overlook sexuality during medical consultations or fail to initiate discussions about sexual activity, especially in older female populations.^44,49-52^ Time constraints, inadequate knowledge and training, complexity of patient comorbidities, and age and sex discordance are contributing factors, but lack of comfort with and preconceptions about sexuality in later life are also significant hurdles.^48,50^ This highlights the need to improve training and communication in sexual health, particularly as a recent study revealed that actively addressing sexuality in gynecological consultations increased the number of diagnosis of sexual problems in postmenopausal women from 12% to 48%.^42^

Lack of confidence in pharmaceutical approaches for sexual dysfunction and fears about side effects of drugs have also been reported.^37,53,54^ Research shows that 6% to 13% of older men and less than 1% of older women use medication to enhance sexual function.^31^ Similarly, there are concerns among many women and HCPs about the perceived risks of systemic HT for VMS and menopause-associated sexual dysfunction.^55-58^ One in eight women in our study experienced sexual problems, but only 2% referred to HT in the free-text data. Much of the controversy around HT stems from concerns about the safety profile of HT including increased risks of cardiovascular disease and breast cancer, which led to an abrupt decline in prescriptions worldwide.^59-62^ Subsequent research and analysis of the early data has shown that risks vary by dosage, regimen, and timing of initiation of HT, and that for most women the benefits outweigh the risks.^63-65^ This is reflected in current guidelines and recommendations, which suggest that HCPs should feel confident in offering systemic HT to most women after an appropriate assessment, using an informed and shared decision-making process.^66,67^

Low-dose vaginal estrogen therapy, however, should also be considered in the treatment of menopause-associated sexual dysfunction, especially for women with vaginal symptoms only or those in whom systemic preparations are contraindicated. The purpose of this treatment is to deliver estrogen directly to the vaginal walls to reduce symptoms through estrogen therapy to the area, with little to no systemic exposure.^68^ It is generally well tolerated and the preferred pharmacological treatment of symptomatic genitourinary syndrome of menopause (GSM), in particular vaginal dryness and dyspareunia secondary to vulvovaginal atrophic changes.^68-70^ Early initiation is recommended, and benefits of long-term use include sustained relief of GSM symptoms as well as physiological improvements.^71^ Multiple vaginal estrogen products (creams, pessaries, or tablets) are available and the choice is determined predominantly by patient preference.

The following design limitations should be considered when interpreting the findings of the present study. Our study sample may not be representative of all women as 98% were white and more research is needed in other racial and ethnic groups. In addition, the results are based on the qualitative data from a “self-selecting” subsample of the UKCTOCS participants. It only mirrors the experiences of those women who were motivated to make comments on the questionnaire, so they may not be illustrative of the larger study population. Further analysis showed that women who left comments were slightly older and less likely to have an intimate partner, or be sexually active.

Attitudes of older women who have infrequent sex (every few months) or experienced changes in function during their participation in UKCTOCS have also not been captured because baseline data (one time-point) were used, and only sexual activity that had taken place in the preceding month was assessed. Lastly, previous studies have shown that as women age, vaginal intercourse declines (from 51% in women in their 50s to 21% in those over 70), and alternative forms of expressing sexuality such as intimate touching or mutual masturbation become more important relative to penetrative sex.^27,54,72^ The FSAQ's focus on sexual intercourse may not accurately reflect what constitutes satisfying sex in this older female population and possibly contribute to “falsely” lower scores. A recent paper confirmed that existing scales for sexual function fail to provide a nuanced picture of sexual experiences in older women and emphasized that new measures are needed to assess the multiple aspects of sexuality in older people.^73^

## CONCLUSIONS

The UKCTOCS data provided a unique opportunity to examine first-hand perspectives on sexual functioning in a large sample of postmenopausal women, illustrating how intimate relationships, health, and psychological factors impact sexual intimacy and satisfaction. Our findings have implications for clinical practice and not only show that sexual activity in older women is multifactorial, but also that sexual difficulties are often underreported, underrecognized and undertreated. The results highlight the need to proactively address sexual functioning in postmenopausal women from a balanced perspective and individualized basis. Open communication about sexuality, including desires, needs, and dysfunctions, is important and will reduce the threshold for women to discuss sexual function.^74^ Additional sexual education for HCPs is required to facilitate this process. Future improvements in sexual health care will help to meet the needs of a growing ageing population, and will provide support to maintain sexual well-being in later life.

## Supplementary Material

Supplemental Digital Content
